# Myeloperoxidase-immunoreactive cells are significantly increased in brain areas affected by neurodegeneration in Parkinson’s and Alzheimer’s disease

**DOI:** 10.1007/s00441-017-2626-8

**Published:** 2017-05-02

**Authors:** Sandra Gellhaar, Dan Sunnemark, Håkan Eriksson, Lars Olson, Dagmar Galter

**Affiliations:** 1 Department of Neuroscience, Karolinska Institute, Retzius Väg 8, 17177 Stockholm, Sweden; 2 AstraZeneca R&D, Södertälje, Sweden; 3 Department of Clinical Neuroscience, Karolinska University Hospital, Stockholm, Sweden

**Keywords:** Frontal cortex, Hippocampus, Substantia nigra, In situ hybridization, Neuroinflammation

## Abstract

Myeloperoxidase (MPO) is a key enzyme in inflammatory and degenerative processes, although conflicting reports have been presented concerning its expression in the brain. We studied the cellular localization of MPO and compared numbers of MPO cells in various brain regions between neurologically healthy individuals and patients with Parkinson’s disease (PD) or Alzheimer’s disease (AD; *n* = 10–25). We also investigated two rodent PD models. MPO immunoreactivity (ir) was detected in monocytes, perivascular macrophages and amoeboid microglia in the human brain parenchyma, whereas no co-localization with glial fibrillary acidic protein (GFAP) ir was observed. In the midbrain, caudate and putamen, we found a significant increase of MPO-immunoreactive cells in PD compared with control brains, whereas in the cerebellum, no difference was apparent. MPO ir was detected neither in neurons nor in occasional small beta-amyloid-immunoreactive plaques in PD or control cases. In the frontal cortex of AD patients, we found significantly more MPO-immunoreactive cells compared with control cases, together with intense MPO ir in extracellular plaques. In the hippocampus of several AD cases, MPO-like ir was observed in some pyramidal neurons. Neither rapid dopamine depletion in the rat PD model, nor slow degeneration of dopamine neurons in MitoPark mice induced the expression of MPO ir in any brain region. MPO mRNA was not detectable with radioactive in situ hybridization in any human or rodent brain area, although myeloid cells from bone marrow displayed clear MPO signals. Our results indicate significant increases of MPO-immunoreactive cells in brain regions affected by neurodegeneration in PD and AD, supporting investigations of MPO inhibitors in novel treatment strategies.

**Electronic supplementary material:**

The online version of this article (doi:10.1007/s00441-017-2626-8) contains supplementary material, which is available to authorized users.

## Introduction

Myeloperoxidase (MPO), a lysosomal enzyme composed of two heavy and two light subunits (Nauseef et al. 1988), is a major constituent of the azurophilic granules of neutrophil granulocytes and is also found in the lysosomes of monocytes (Nichols and Bainton 1973). MPO catalyzes, in the presence of chloride ions, the conversion of hydrogen peroxide to hypochlorous acid (HOCl), which then reacts with proteins and other biological species to form oxidation products, e.g., 3-chlorotyrosine protein adducts (Domigan et al. 1995; Harrison and Schultz 1976; Zgliczynski et al. 1968). MPO activity plays a crucial role not only in the physiology of host defense against microorganisms but also in the pathophysiology of cardiac dysfunction, atherosclerosis, respiratory tract and central nervous system diseases (Malle et al. 2007; Nathan 2006). Increased serum levels of MPO are predictors of major adverse cardiac events (Baldus et al. 2003) and have recently also been described in patients with Alzheimer’s disease (AD; Tzikas et al. 2014). In addition, correlations between increased serum MPO levels and reduced cognitive functions have been demonstrated in recurrent depressive disorder (Talarowska et al. 2014).

In brain tissue, increased levels of MPO have been reported in several neurodegenerative disorders. In Parkinson’s disease (PD) and Huntington’s disease (HD) significantly higher MPO protein levels have been revealed in the midbrain and in caudate nucleus samples, respectively, whereas in amyotrophic lateral sclerosis (ALS), no differences have been detected in motor cortex samples compared with control cases (Choi et al. 2005). In AD, increased MPO levels have been reported in the frontal cortex (Green et al. 2004; Reynolds et al. 1999) localized mainly in amyloid beta (Aβ)-positive senile plaques and in some activated microglia. In addition, neuronal expression of MPO has been described in AD brain areas including in the granular and pyramidal neurons of the hippocampus. By contrast, MPO expression has been localized to astrocytes in the vicinity of dopamine neurons in PD midbrain samples and in a PD mouse model based on the neurotoxin 1-methyl-4-phenyl-1,2,3,6-tetrahydropyridine (MPTP; Choi et al. 2005; Chung et al. 2010). In addition, the induction of MPO mRNA has been demonstrated in a mouse hippocampal cell line and in ventral midbrain samples from MPTP-treated mice by using quantitative reverse transcription plus the polymerase chain reaction (RT-PCR; Choi et al. 2005; Green et al. 2004), whereas several other studies have identified MPO gene activity only in blood-derived cells of the myeloid linage (Chang et al. 1986; Reynolds et al. 2006).

We aim at clarifying the cellular localization of MPO in brain tissue from patients with neurodegeneration and compare numbers of MPO cells in a large number of patients with those in age- and sex-matched control cases. We observe a significant increase in MPO-immunoreactive cells in human brain areas affected by neurodegeneration, a finding that supports recent clinical trials with a MPO inhibitor to improve PD treatment (Jucaite et al. 2015). In the two analyzed rodent PD models, we did not detect any increase in MPO expression in the brain.

## Materials and methods

### Human postmortem tissue

Age- and sex-matched human postmortem AD, PD and control brain samples were provided by The Netherland Brain Bank, Harvard Brain Tissue Resource Center (Belmont, USA) and the Queen Square Brain Bank for Neurological Disorders, London and were selected based on neuropathological diagnosis. For PD, we included the striatum, ventral midbrain and cerebellum and, for AD, we included frontal cortex and hippocampus samples. Relevant clinical information regarding all cases is compiled in Table 1. The brain material that we analyzed was mainly from advanced cases of the two neurodegenerative diseases. Nineteen of the 25 AD cases studied were scored as AD Braak 5 or Braak 6, whereas for the remaining six cases, we had no information. Similarly for the PD cases, we deduced from the small number of remaining dopamine neurons in midbrain sections that most cases were end-stage cases. We used mainly fresh frozen tissue to enable the analysis of the same brain samples with two techniques, namely immunohistochemistry and in situ hybridization. Cryosections of 14 μm were mounted on SuperFrost Plus microscope slides (Microm, Germany) and stored at −20 °C until processed for analysis. To test the specificity of the MPO antibodies, we used spleen tissue from two control cases provided by the University of Maryland Brain and Tissue Bank (USA; Supplemental Fig. 1). Sections from paraformaldehyde-fixed tissue samples of the frontal cortex and hippocampus of several control subjects with no neurological disease were analyzed by the same histological staining protocols in order to compare antibody staining with previously published data (Supplemental Fig. 1).

**Table 1:** Clinical and neuropathological characteristics of autopsy cases. No significant differences were detected between patients and control groups for the listed variables (*PD* Parkinson disease, *AD* Alzheimer disease, *SEM* standard error of the mean, *PMI* post-mortem interval [hours from death to freezing of specimen])

Diagnosis	Brain area	Number	Age (years ± SEM)	Sex (male + female)	PMI (hours ± SEM)
PD	Putamen	10	81.4 ± 6.7	7 + 3	14:58 ± 7:20
Controls	Putamen	10	76.4 ± 9.1	7 + 3	16:26 ± 2:54
PD	Caudate nucleus	10	81.4 ± 6.7	7 + 3	14:58 ± 7:20
Controls	Caudate nucleus	10	76.4 ± 9.1	7 + 3	16:26 ± 2:54
PD	Substantia nigra	16	76.3 ± 6.4	12 + 4	12:37 ± 7:46
Controls	Substantia nigra	18	73.4 ± 12.5	12 + 6	14:08 ± 7:18
PD	Cerebellum	10	76.2 ± 5.4	7 + 3	22:50 ± 20:45
Controls	Cerebellum	10	78.7 ± 8.9	5 + 5	24:53 ± 22:09
AD	Frontal cortex	25	76.4 ± 11.9	12 + 13	5:30 ± 1:40
Controls	Frontal cortex	20	76.3 ± 11.8	9 + 11	6:42 ± 1:32
AD	Hippocampus	25	74.7 ± 11.3	13 + 12	8:01 ± 4:52
Controls	Hippocampus	20	57.1 ± 11.8	10 + 10	9:10 ± 4:48

### Animal tissue and PD animal model

Twenty-four adult female Sprague-Dawley rats were kept under standard laboratory conditions: four per cage with free access to food and water. Lesions were achieved by stereotactic injection of 6-hydroxydopamine (6-OHDA; Sigma, Sweden: 2 mg/ml in 0.9% saline with 0.2 mg/ml ascorbic acid, at the coordinates 4.4 mm posterior and 1.2 mm lateral to bregma and 7.8 mm below the dura mater under isoflurane anesthesia). At 12 h, 24 h, 48 h, 7 days and 3 weeks after operation, four animals from different cages were killed and their brains and spleens were dissected, frozen on dry ice and kept at −80 °C until use. The femur bones from four rats were dissected and bone marrow was flushed out with phosphate-buffered saline (PBS). Following several washing steps, cell smears were prepared and air-dried. At 12 and 24 h following toxin injection, two further rats were trans-cardially perfused with LANA’s fixative (4% paraformaldehyde, 14% picric acid) and their brains and spleens were processed for cryoprotection and cryosectioning.

MitoPark mice of 12-, 20- and 28-weeks-of-age were included in the study, representing early, moderate and late stages of striatal DA depletion. Five animals per age group were killed and their brains were frozen on dry ice and cryosectioned as described for rat and human tissue. Sections from the striatum and midbrain were used for immunohistochemistry or in situ hybridization.

### Immunohistochemistry

The tissue sections were dried at room temperature before treatment with LANA fixative for 10 min. Incubation in hydrogen peroxide solution (2% H_2_O_2_ in 70% methanol) for 10 min blocked endogenous peroxidase activity and goat serum (5%, 60 min) reduced unspecific binding. Treatment with primary antibody was carried out at 4 °C, typically overnight or for 32 h and the binding of the antibody was visualized with the chromogenic substrate 3,3′-diaminobenzidine (DAB) after incubation with the respective secondary biotin-labeled antibody. The sections were counterstained with cresylviolet, dehydrated and mounted with Entellan (VWR, Sweden) for microscopy (Axiophot Zeiss, with AxioVision software, Zeiss). Negative controls, in which the primary antibody was omitted, were included with each immunostaining and at least one section of each tissue specimen was also analyzed as a negative control. Sections from post mortem spleen, in which abundant neutrophils and monocytes were present, were used as positive controls (Supplemental Fig. 1a). To test the specificity of the MPO antibodies further, we performed Western blot with protein extracts from spleen and detected the expected light and heavy bands. The heavy MPO band was partially glycosylated resulting in further bands (Supplemental Fig. 1b).

The following primary antibodies were used for studies of human tissues: polyclonal rabbit anti-MPO antibodies (Biodesign K51891R, MediQip Sweden 1/200, previously reported by Green et al. 2004; Dako Cytomation, Sweden, 1:600, previously reported by Choi et al. 2005), monoclonal anti-PHF-Tau antibody (Clone AT8, Innogenetics, Belgium), monoclonal anti-Aβ protein (Clone 6E10, Chemicon, Sweden, 1:1000), anti-glial fibrillary acidic protein (GFAP) antibodies (polyclonal rabbit and mouse clone G-A-5, Sigma, USA), histocompatibility antigen (HLA)-DR (HLA-DR), alpha chain, mouse clone 1B5 expressed by microglia (Dako Cytomation, 1:100), mouse monoclonal anti-human CD68 (clone PG-M1, Serotec, MediQip, Sweden, 1/200, a cell surface glycoprotein expressed by macrophages and monocytes) and polyclonal 3-chlorotyrosine antibody (Hycult biotech, Nordic Biosite, Sweden, 1:150).

For the rodent animal models, rabbit polyclonal anti-MPO antibodies (Lab Vison, Ah-diagnostic, Sweden, 1:500; Thermo, USA) were used to detect MPO and mouse monoclonal anti-rat integrin M (CD11b; clone OX-42) and CD68 (clone ED1, Chemicon, Sweden, 1:200) to detect microglia.

### Quantification of MPO immunoreactivity

For each brain sample, we analyzed three to four sections at approximately 30 to 50 section intervals (0.4 to 0.6 mm) for MPO immunoreactivity (ir). One or two additional sections were used as negative controls (omitting the primary antibody). The MPO-immunoreactive cells were counted on ten non-overlapping optical fields by using a 10× lens in the caudate nucleus and putamen, in layers one to six of the frontal cortex and in the molecular, Purkinje and granular layers of the cerebellum. For midbrain sections, all MPO-immunoreactive cells detected in 10 non-overlapping optical fields including at least one neuromelanin-positive dopamine neuron were counted. The scatter plots show the average number of MPO cells per millimeter squared for each brain sample. The unpaired double-sided Student’s *t*-test was used for statistical analysis (Prism 5, GraphPad, San Diego, Calif., USA).

### In situ hybridization

**Fig. 1:**
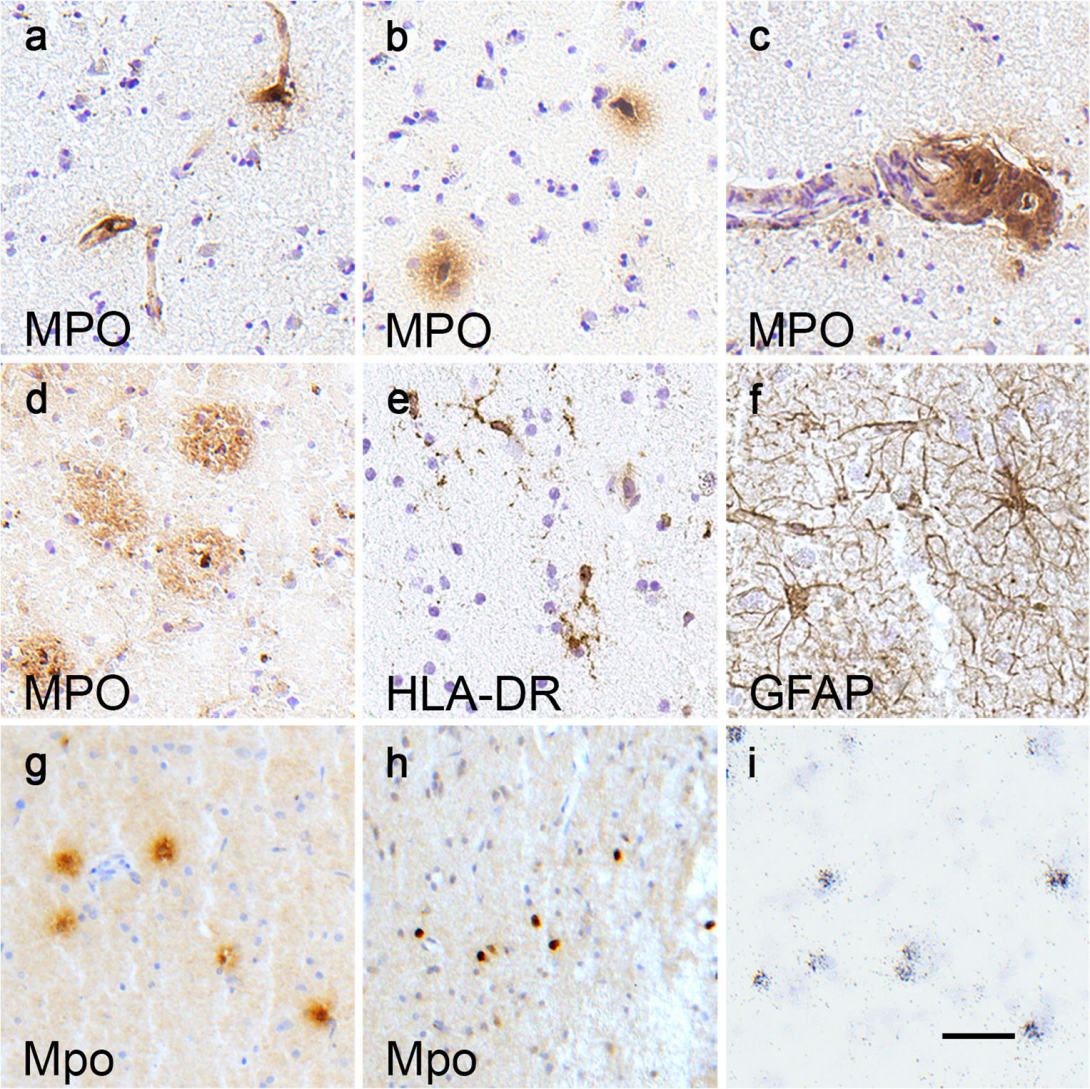
Typical cellular localization of myeloperoxidase (*MPO*) expression in human and rodent brain tissues. In all brain areas, analyzed cells with MPO immunoreactivity (ir) were often located in small blood vessels (**a**) or in the brain parenchyma close to blood vessels (**b**) and, in some cases, in the wall of larger blood vessels (**c**). In brain tissue from AD patients, MPO ir was also detected in amyloid plaques and round cells with MPO ir were occasionally found close to the extracellular protein aggregates (**d**). Adjacent brain sections were stained for histocompatibility antigen (*HLA-DR*; **e**) and glial fibrillary acidic protein (*GFAP*; **f**) to reveal the shape and size of ramified microglia and astrocytes, respectively. Examples of MPO-immunoreactive cells are taken from the hippocampus of a control case (**a**, **c**), the caudate nucleus of a case with Parkinson’s disease (PD; **b**) and the hippocampus of a case with Alzheimer’s disease (AD; **d**). The sections showing HLA and GFAP staining were taken adjacent to sections from the caudate nucleus of the PD case in **b**. In rat brains, cells with MPO ir were only detected at the site of toxin injection 24 h after operation, both in fresh frozen rat brain tissue (**g**) and in brain tissue from perfusion-fixed rats (**h**). In fresh frozen tissue, MPO-positive cells were often surrounded by a halo of ir, whereas in tissue from animals perfused with fixative solution, the ir was restricted to cell boundaries. In rat bone marrow smears, MPO mRNA was readily detected by radioactive in situ hybridization (**i**). *Bar* 25 μm

In situ hybridization was performed as described earlier (Westerlund et al. 2008) with species-specific oligonucleotide probes complementary to MPO. Briefly, ^33^P-dATP-labeled probes with a length of 50 nucleotides were hybridized overnight to cryosections, which were then washed, dehydrated and exposed to autoradiographic films (Biomax, Kodak) or dipped in photo-emulsion, developed after 3 weeks and inspected under a light microscope following counterstaining with cresylviolet. The following oligonucleotides targeting various exons of the gene were used: for human MPO ref.|NM_000250.1| nt = 820–771, nt = 1719–1672, nt = 1898–1851, and nt = 2247–2198; for rat ref.|NM_001107036.1| nt = 585–538, nt = 1170–1126, nt = 1349–1300, and nt = 1361–1314; for mouse MPO ref.|NM_010824.2| nt = 716–669, nt = 1758–1717, and nt = 1949–1900. The probe-signal shown in Fig. 1i corresponds to nt = 1349–1300.

## Results and discussion

We examined MPO expression in the nervous system and found that, in all brain areas analyzed, the typical MPO-immunoreactive cells were small, had a round or slightly elongated shape and were located in or in close proximity to blood vessels (Fig. 1a, b). In some cases, particularly in areas with extensive neurodegeneration, MPO ir was detected in the wall of larger blood vessels, including in round cells in the vessel wall (Fig. 1c). In brain tissue from AD patients, extracellular MPO ir was also detected in amyloid plaques and round MPO-immunoreactive cells were occasionally found close to these protein aggregates (Fig. 1d), confirming earlier reports (Reynolds et al. 1999). Adjacent brain sections were stained for HLA (Fig. 1e) and GFAP (Fig. 1f) to reveal the shape and size of ramified microglia and astrocytes, respectively. MPO was not expressed by astrocytes or ramified microglia, as assessed by double-immunostaining with the respective markers (data not shown), in contrast to the findings reported in human midbrain samples from PD patients (Choi et al. 2005). Moreover, in our rodent animal models of PD, we did not detect any MPO expression in astrocytes, in contrast to the findings in the MPTP mouse model (Choi et al. 2005; Chung et al. 2010). In multiple system atrophy (MSA), a neurodegenerative α-synucleinopathy with intracellular aggregations in oligodendrocytes, induction of MPO expression was demonstrated in activated microglia but not in astrocytes in human brain and in a mouse model of MSA (Stefanova et al. 2012). Treatment with MPO inhibitors was shown effectively to suppress microglia activation in the MSA mouse model and to protect against neuronal loss in early non-symptomatic stages but not after the onset of full-blown pathology in late stages (Kaindlstorfer et al. 2015; Stefanova et al. 2012).

In the PD mouse model MitoPark, the slow degeneration of dopamine neurons did not induce MPO expression in either astroglia or microglia cells. A few MPO-immunoreactive cells were detected in rat brains at the site of 6-OHDA toxin injection at 24 h after treatment (Fig. 1g). These MPO-immunoreactive cells were probably infiltrated blood cells, similar to those involved in the inflammatory cell infiltration demonstrated in human and rat spinal cords after injury (Abrams et al. 2012; Fleming et al. 2006). At all later time points analyzed (3, 7, and 21 days after toxin injection), MPO expression was not detectable in the striatum, in the midbrain, or at the injection site. We also compared the morphology of MPO-immunoreactive cells in fresh frozen rat brain tissue (Fig. 1g) and in brain tissue from rats perfused with fixative (Fig. 1h) at 24 h after toxin injection. In fresh frozen tissue, MPO-immunoreactive cells were often surrounded by a halo of immunostaining, whereas in tissue from animals perfused with fixative, MPO ir was restricted to cell boundaries. The halo detected in fresh frozen brain tissue is therefore possibly an artifact attributable to leaky MPO vesicles, perforated by crystal formation during the freezing process. To test this hypothesis, we examined formalin-fixed human brain samples and found that staining for MPO generated similar results as in the perfused rat brain, namely round cells with no surrounding halo (Supplementary Fig. 1c).

Next, we used radioactive in situ hybridization in an attempt to localize MPO mRNA in human brain and in tissues from animal models of neurodegeneration. In rat bone marrow, MPO mRNA was readily detected by our method (Fig. 1i), whereas no signal was found in any human or rodent brain tissue analyzed. Our findings are compatible with the hypothesis that substantial MPO transcription occurs during the development of myeloid cells in the bone marrow (Chang et al. 1986), whereas circulating cells in the blood stream or infiltrating cells in the brain parenchyma have very low levels of MPO transcription, below the detection limits of our methods.

Our results also demonstrate the limitations of rodent animal models to mimic all aspects of human brain diseases. The dopamine depletion in target brain areas is faithfully reproduced by both models but other pathological changes such as the interaction between immune and nervous systems are less-well modeled. In addition to the immune systems of humans, mice and rats being quite different, we need to take into account the fact that laboratory animals are kept in sterile environments and that their immune systems are thus less challenged than those of humans or non-laboratory animals. This might contribute to the absence of immune response such as the presence of MPO-positive cells in models of neurodegeneration.

### MPO expression in PD

**Fig. 2:**
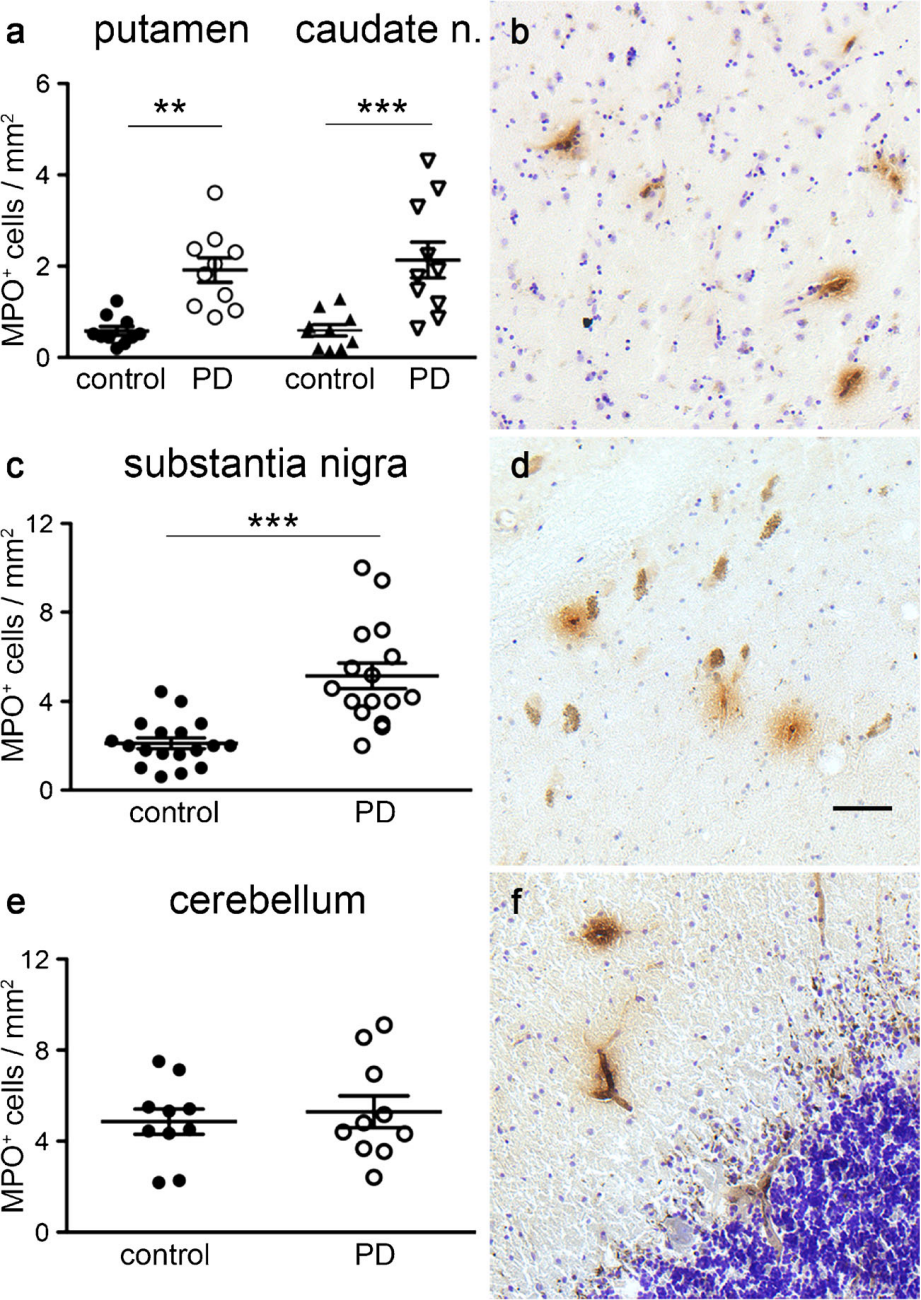
Increased MPO expression in PD restricted to brain areas affected by neuropathology. Numbers of MPO-immunoreactive cells are significantly higher in the putamen and caudate nucleus from PD patients compared with controls (*P* < 0.001, *t* = 4.66, df = 18; *P* = 0.001, *t* = 3.72, df = 18, respectively, in **a**). Examples of MPO-immunoreactive cells are shown in the putamen of a PD case (**b**). The number of MPO-immunoreactive cells was significantly higher in the substantia nigra of PD patients compared with controls (*P* < 0.001, *t* = 5.10, df = 32, in **c**). Examples of MPO-immunoreactive cells are shown in the midbrain of PD patients, close to the remaining neuromelanin-containing dopamine neurons (**d**). The number of MPO-immunoreactive cells in the cerebellar cortex of PD patients did not differ from that of control cases (*P* > 0.05, in **e**). Examples of MPO-immunoreactive cells are shown in the cerebellar cortex of a PD patient (**f**). *Bar* 25 μm. **P < 0.01, ***P < 0.005

In agreement with earlier studies that quantified MPO protein levels in brain tissue, we found a marked difference in the numbers of MPO-positive cells between controls and PD cases in three different brain areas (Fig. 2). In the putamen, we counted an average number of 0.59 ± 0.10 MPO-positive cells per mm^2^ in controls and 1.92 ± 0.27 in PD, leading to a statistically significant difference in the unpaired two-tailed *t*-test (*P* < 0.001, *t* = 4.66 and df = 18; Fig. 2a). In the caudate nucleus, we found an average number of 0.60 ± 0.13 MPO-positive cells per mm^2^ in controls and 2.13 ± 0.39 such cells per mm^2^ in PD, leading to a statistically significant difference in the unpaired two-tailed *t*-test (*P* = 0.001, *t* = 3.72 and df = 18). Examples of MPO-immunoractive cells in the putamen are shown in Fig. 2b.

Next, we analyzed midbrain sections from PD cases and matched control cases. Again, the MPO ir was confined to cells with a round or slightly elongated form and many appeared to be located close to or in blood vessels. Contrary to earlier findings reporting MPO expression in astrocytes close to remaining DA neurons in PD patients and in the midbrain of MPTP treated mice (Choi et al. 2005), we did not detect any MPO-immunoreactive astrocytes in any of the 15 PD brains analyzed. In addition, we analyzed midbrains from two rodent PD models in detail and did not detect any MPO expression in astrocytes (data not shown). Examples of MPO-positive cells amongst surviving mesencephalic dopamine neurons in a Parkinsonian brain are shown in Fig. 2d. The number of MPO-immunoreactive cells differed significantly between PD (means ± SEM: 5.16 ± 0.57, *n* = 16) and control (2.11 ± 0.24, *n* = 18; two-tailed *t*-test: *P* < 0.001, *t* = 5.1, df = 32; Fig. 2c) cases. The large difference in cell numbers between PD patients and controls is probably the reason for the reported difference in MPO protein levels, rather than an induction of MPO in astrocytes, as has been suggested (Choi et al. 2005). The absence of detectable MPO mRNA expression in the midbrain of PD patients (data not shown) supports the view that the increased MPO level stems from inflammatory cells rather than being induced in astrocytes in the midbrain. In addition, we did not detect any MPO protein or mRNA induction in the two rodent PD models, either in the striatum or in the midbrain. In the unilateral 6-OHDA rat model, we found MPO ir in brain tissue around the toxin injection site (Fig. 1g, h), whereas the activated microglia in the striatum showed no MPO ir (not shown). The midbrain astrocytes did not express any detectable levels of MPO, unlike the findings in the MPTP mouse model (Choi et al. 2005). Rotenone, a further toxin known to induce PD symptoms in rodents, has been shown to induce MPO mRNA and protein expression in cultured microglial cells (Chang et al. 1986) but in vivo induction has not been analyzed. In MitoPark mice, a genetic PD model with a slow progression of the degeneration of DA neurons and nerve terminals, we did not detect enhanced microglia activation in the striatum or MPO induction in astrocytes in the midbrain (data not shown). Three different ages of MitoPark mice were analyzed, with different stages of striatal DA depletion, in order to reflect early, moderate and late stages of PD.

To test whether the number of MPO-immunoreactive cells increased in all brain areas in PD patients, we also analyzed the cerebellum. Only areas of the cerebellar cortex were included in the analysis because the density of blood vessels varies greatly between gray and white matter. No difference in the number of cells with MPO ir was found between PD and control cases (4.86 ± 0.55 versus 5.29 ± 0.70, two-tailed *t*-test *P* = 0.63, *t* = 0.48, DF = 18; Fig. 2e). Examples of MPO-immunoreactive cells are shown in Fig. 2f. Our results indicate that MPO expression is not increased in the whole brain in PD and that the number of MPO cells is elevated primarily in brain areas affected by pathological processes, such as the striatum and substantia nigra in PD.

### MPO expression in AD

Two brain areas with severe AD pathology were analyzed, namely the frontal cortex and anterior hippocampus.

**Fig. 3:**
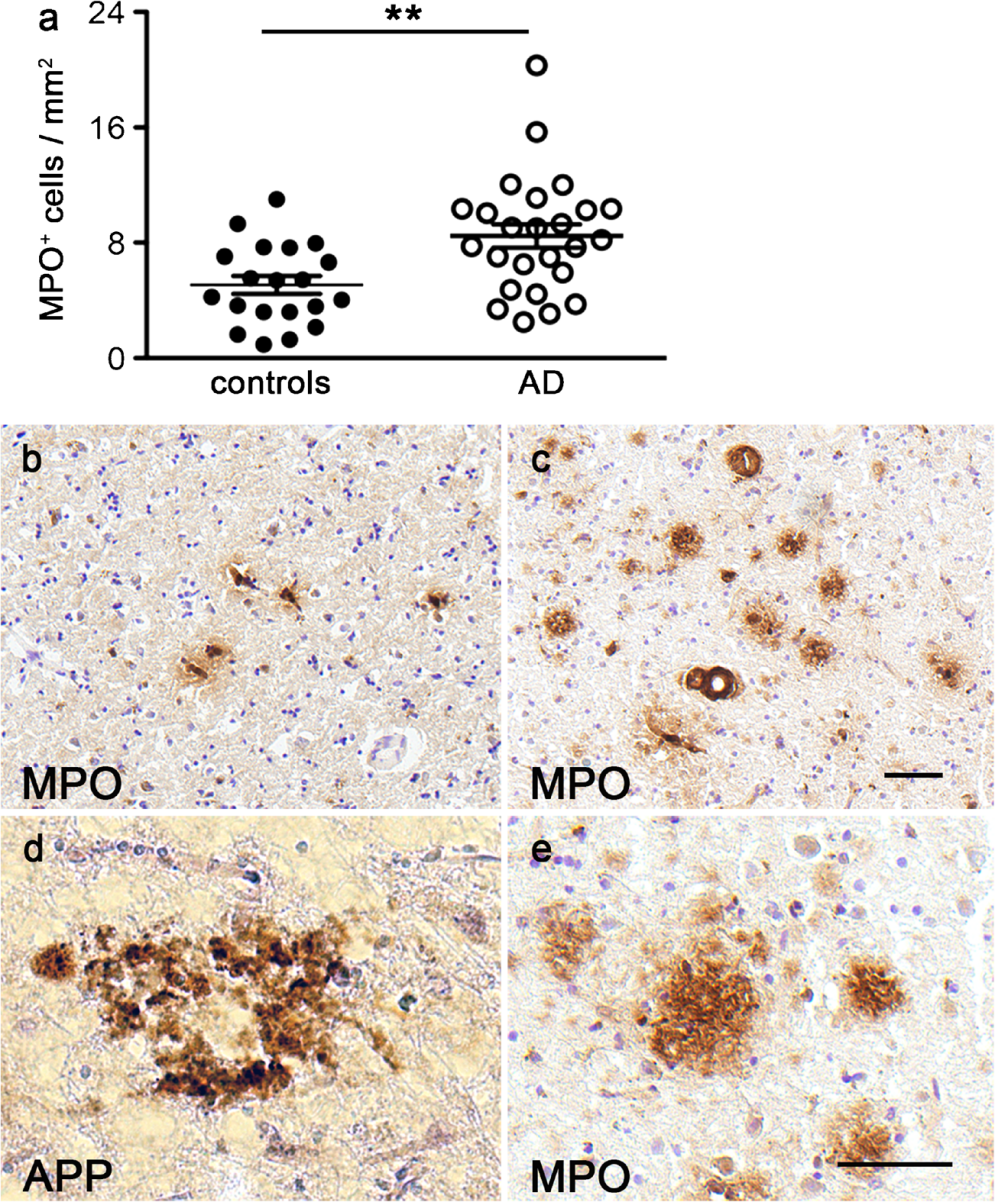
Increased MPO expression in frontal cortex samples from AD patients. The number of MPO-immunoreactive cells was significantly higher in the frontal cortex of AD patients compared with controls (*P* = 0.003, *t* = 3.17, df = 63, in **a**). Examples of MPO-immunoreactive cells are shown in the frontal cortex of a control case (b) and an AD patient (**c**). Many extracellular protein aggregates show immunostaining for amyloid beta (*APP*, **d**) and also for MPO (**e**) in samples from AD patients. *Bars* 25 μm (**b**, **c**; **d**, **e**). ***P < 0.005

The number of cells with MPO ir in the frontal cortex was significantly increased in AD cases (means ± SEM 8.46 ± 0.81, *n* = 25) compared with control cases (5.08 ± 0.62, *n* = 20), leading to a highly significant difference in the two-tailed *t*-test (*P* = 0.003, *t* = 3.17, df = 43; Fig. 3a). Examples of MPO-immunoreactive cells in the cortex from a healthy control case and an AD patient (Fig. 3b, c) showed an increase in enzyme expression, often in proximity to blood vessels. Vascular and systemic inflammation and cerebrovascular alterations have recently been recognized as important contributing factors to the pathophysiology of AD and as possible new treatment targets (Takeda et al. 2014). Our findings in brain tissue are in agreement with the recently reported significantly increased MPO plasma levels detected in patients with AD and the association between MPO plasma levels and the Aβ_1–42/1–40_ ratio (Tzikas et al. 2014). An earlier study compared plasma levels of several hundreds of proteins or peptides between patients with AD, mild cognitive impairment (MCI), or other dementias and age-matched control cases by using multianalyte profiling. However, this report failed to identify an association between MPO and AD, possibly because of the mixed patient group studied (Hu et al. 2012). In this respect, we feel it important to point out that the brain material that we analyzed was mainly from advanced cases of AD and thus, the results might be very different in MCI or early cases of AD.

**Fig. 4:**
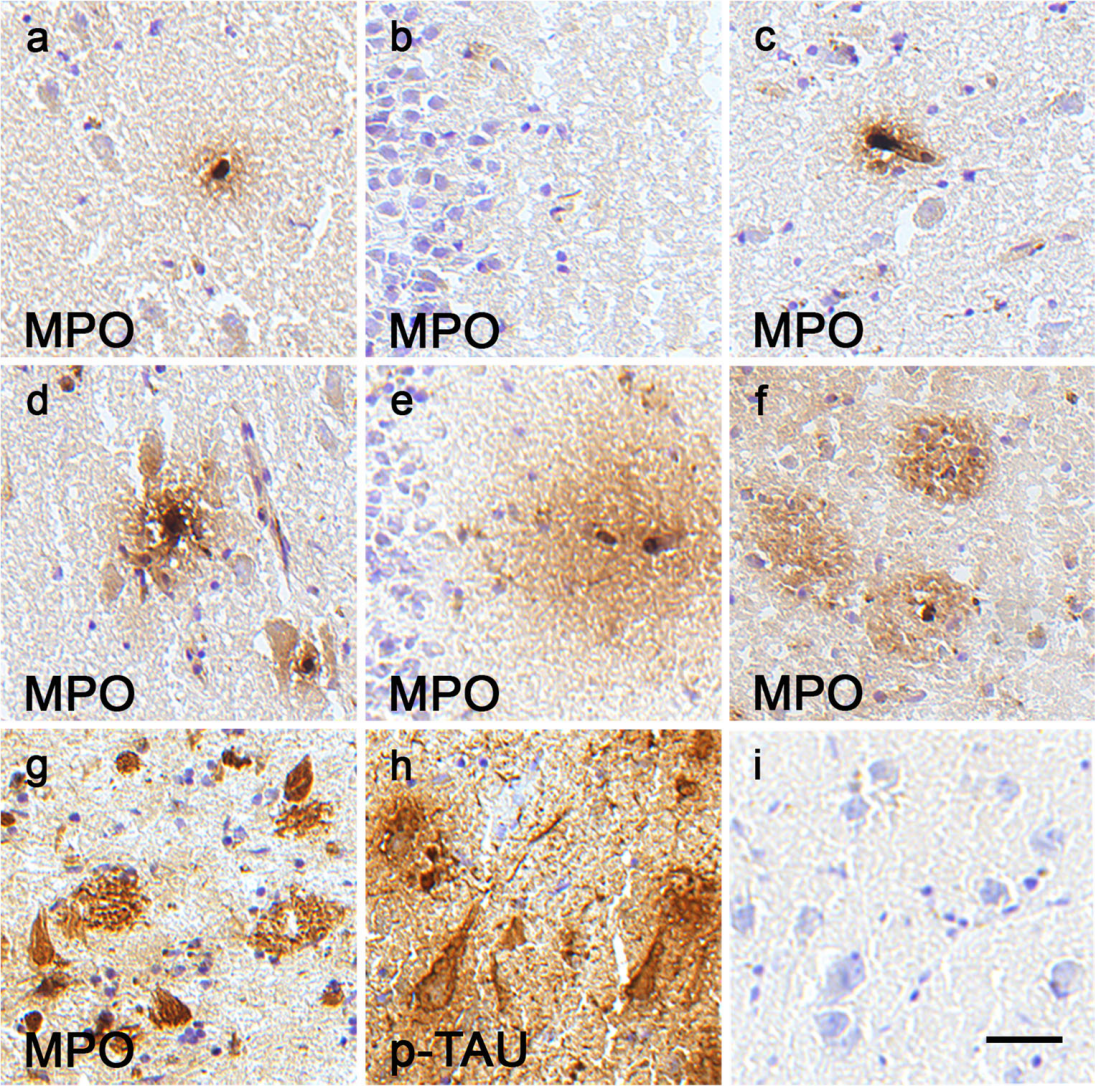
MPO expression in hippocampus from AD patients and control cases. Examples of MPO-immunoreactive cells in the CA1 region (**a**), dentate gyrus (**b**) and entorhinal cortex (**c**) of a control case and in the CA1 region (**d**), dentate gyrus (**e**) and entorhinal cortex (**f**) of an AD patient. Some “ghost tangles” are shown scattered between MPO-immunoreactive amyloid plaques in the CA1 region of an AD case (**g**). Similar cells on the adjacent section are ir for tau (*p-TAU*; **h**). A negative control for the staining protocol is shown in **i**. *Bar* 25 μm

In addition to MPO-immunoreactive cells, we detected MPO protein ir attached to many extracellular amyloid plaques in all AD cases (Fig. 3d, e). Co-localization of MPO with Aβ peptides in amyloid plaques, in cortical blood vessels and in CD68 positive microglia-macrophages around plaques has previously been described in AD brain tissue by Reynolds et al. (1999) who also suggested that a specific polymorphism in MPO is associated with a gender-specific risk for AD, increasing the risk in women but not in men. A later study including a much larger amount of Spanish case-control material and a meta-analysis of data from Florida (USA), Finland, Spain and California (USA) failed to find a difference in MPO genotype or allele frequencies between AD and control cases (Combarros et al. 2002). Nevertheless, the quantification of MPO protein levels by Western blot in the frontal cortex from nine control and 14 AD patients demonstrated significantly increased MPO levels in the AD cases (Green et al. 2004), supporting our finding. Furthermore, Green et al. (2004) reported no correlation with age at death for either control samples only or for all samples analyzed. Similarly, we found no correlation between age at death and number of cells with MPO ir in the frontal cortex for control cases (r_s_ = −0.004, *P* = 0.987, *n* = 20) or for all samples (r_s_ = 0.066, *P* = 0.665, *n* = 45). In contrast to reported findings in cultured murine and human neuroblastoma cells and in the temporal cortex of AD patients (Green et al. 2004), we did not detect any neuronal MPO ir in any frontal cortex samples (*n* = 45). We also analyzed hippocampal sections from controls (Fig. 4a–c) and AD patients (Fig. 4d–i). Examples from the CA1 region (Fig. 4a, d), dentate gyrus (Fig. 4b, e) and entorhinal cortex (Fig. 4c, f) demonstrated that MPO-immunoreactive cells were often in or close to small blood vessels. Contrary to the previously reported MPO staining of dentate neurons and pyramidal neurons in AD patients and the weak staining of pyramidal neurons in control cases (Green et al. 2004), we did not detect any neuronal MPO ir. Many amyloid plaques with MPO ir were observed in AD hippocampus (Fig. 4f, g), as has been previously described (Green et al. 2004). In seven of the 25 AD samples analyzed, we detected MPO-immunoreactive structures with the shape of pyramidal neurons without a visible nucleus (Fig. 4g) reminiscent of extracellular “ghost tangles” reported to contain highly modified proteins (Bancher et al. 1989; Duong et al. 1993; Good et al. 1996). Immunostaining with PHF-tau antibody on adjacent sections indicated some overlap between MPO and tau ir (Fig. 4h), suggesting that the MPO ir was in part attached to highly phosphorylated proteins. The same distribution of the ir was previously detected with two different MPO antibodies in post mortem human studies (Green et al. 2004), whereas very little signal was detected when we omitted the primary antibody (negative control, Fig. 4i).

To test the hypothesis that the MPO ir seen in AD brain samples around and in plaques is enzymatically active MPO secreted from cells not residing in the brain parenchyma, we incubated sections with antibodies recognizing 3-chlorotyrosine protein adducts. These protein modifications are reliable biomarkers of MPO-induced cytotoxicity in vivo and have been detected with the same antibody in liver samples from well-documented liver injuries (Gujral et al. 2004). No chlorotyrosine protein adducts were detected in brain tissue from humans or rodent animal models, despite a clearly detectable signal on cortex sections treated with hypochloric acid as a positive control (not shown). Increased levels of 3-chlorotyrosine proteins have been reported earlier in AD hippocampus when analyzed by solid-phase chromatography and mass spectrometry (GC/MS method; Green et al. 2004). The lower detection limit of this method might explain the discrepancy between our results and the published study. Furthermore, we used radioactive in situ hybridization to compare MPO mRNA expression between patients and controls and found that none of the cell types showing MPO ir in the brain expressed detectable mRNA levels (data not shown).

In summary, our results indicated that the number of MPO-immunoreactive cells in the frontal cortex of AD patients was increased compared with control cases and that these cells were most likely blood-derived immune cells.

### Concluding remarks

The numbers of MPO-immunoreactive cells are significantly increased in brain areas affected by neurodegeneration, such as the frontal cortex in AD and the caudate, putamen and midbrain in PD. These cells did not resemble or co-localize with markers for astrocytes or neurons but were confined to microglia and blood-derived cells. Our results thus highlight the inflammatory component of the pathophysiology of these neurodegenerative diseases. Combined with studies showing that ablation or inhibition of MPO reduces the neurodegenerative PD phenotype (Choi et al. 2005; Chung et al. 2010; Huh et al. 2011), our findings suggest the exploration of MPO inhibitors in the treatment of neurodegenerative disorders. However, notably, we analyzed late stages of the diseases; the immune response in early stages of neurodegeneration might be different and should therefore be evaluated accordingly.

## Electronic supplementary material

Supplementary Figure 1 MPO expression in immune cells of the spleen and on paraformaldehyde-fixed brain tissue. MPO antibody detected immune cells on fresh frozen sections of human spleen (**a**) and in bands of the heavy and light MPO subunits (around 55 kDa and 12 kDa) in Western blot from spleen lysate (**b**) confirming the specificity of the antibody. Examples of MPO-immunoreactive cells in paraformaldehyde-fixed human brain sections demonstrate the absence of an ir halo around the stained cells in a hippocampal sample from a control case (**c**). *Bar* 25 μm. (GIF 104 kb)

High Resolution image (TIFF 5254 kb)
