# Supplementary figures and images for: Myeloperoxidase-immunoreactive cells are significantly increased in brain areas affected by neurodegeneration in Parkinson’s and Alzheimer’s disease
Source: Cell Tissue Res. 2017 May 2;369(3):445–54. doi: 10.1007/s00441-017-2626-8 (PMC5579172; doi:10.1007/s00441-017-2626-8)

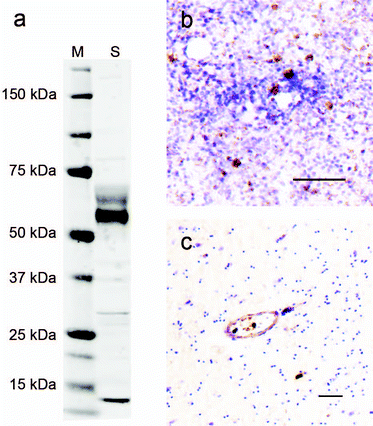

Supplement: Supplementary file 1 — MPO expression in immune cells of the spleen and on paraformaldehyde-fixed brain tissue. MPO antibody detected immune cells on fresh frozen sections of human spleen (a) and in bands of the heavy and light MPO subunits (around 55 kDa and 12 kDa) in Western blot from spleen lysate (b) confirming the specificity of the antibody. Examples of MPO-immunoreactive cells in paraformaldehyde-fixed human brain sections demonstrate the absence of an ir halo around the stained cells in a hippocampal sample from a control case (c). Bar 25 μm. (GIF 104 kb) [file 441_2017_2626_Fig5_ESM.gif]
